# The Pathogenesis of Ossification of the Posterior Longitudinal Ligament

**DOI:** 10.14336/AD.2017.0201

**Published:** 2017-10-01

**Authors:** Liang Yan, Rui Gao, Yang Liu, Baorong He, Shemin Lv, Dingjun Hao

**Affiliations:** ^1^Department of Spine Surgery, Hong Hui Hospital, Xi’an Jiaotong University College of Medicine, Xi’an, 710054, China; ^2^Department of Respiration, The Children’s Hospital of Xi’an City, Xi’an, 710054, China; ^3^Xi’an Jiaotong University College of Medicine, Xi’an, 710054, China

**Keywords:** Heterotopic ossification, OPLL, BMP, TGF-β, Mesenchymal stem cells, MAPK

## Abstract

Ossification of the posterior longitudinal ligament (OPLL) is a multi-factorial disease involving an ectopic bone formation of spinal ligaments. It affects 0.8-3.0% aging Asian and 0.1-1.7% aging European Caucasian. The ossified ligament compresses nerve roots in the spinal cord and causes serious neurological problems such as myelopathy and radiculopathy. Research in understanding pathogenesis of OPLL over the past several decades have revealed many genetic and non-genetic factors contributing to the development and progress of OPLL. The characterizations of aberrant signaling of bone morphogenetic protein (BMP) and mitogen-activated protein kinases (MAPK), and the pathological phenotypes of OPLL-derived mesenchymal stem cells (MSCs) have provided new insights on the molecular mechanisms underlying OPLL. This paper reviews the recent progress in understanding the pathophysiology of OPLL and proposes future research directions on OPLL.

Ossification of the posterior longitudinal ligament (OPLL) was first reported by Key in 1838 [1] and described in detail by Tsukimoto and colleagues in 1960 [2]. OPLL is a disorder characterized by progressive ectopic ossification of the posterior longitudinal ligament (PLL), with occurrence of 70% in cervical spine and 15% in thoracic vertebra as well as in lumbar vertebra [3-5]. The predominant site of OPLL is the cervical segments, with the most frequent level at C5 [3, 4, 6]. Four subtypes of ossification of PLL have been categorized: segmental; continuous; mixed; and localized, circumscribed, or bridged [7]. Patients with OPLL usually develop into various degrees of neurological symptoms from discomfort to severe myelopathy due to compression of the spinal cord and nerve roots by progressively calcified PLL. Epstein *et. al*. estimated that up to 25% patients with cervical myelopathy presenting OPLL [8]. The motility and life quality of patients are greatly affected by these symptoms.

Most of OPLLs occur after age 40, with an average onset age of 50 [9]. OPLL occurs predominantly in male, nearly twice as often as in female. The overall prevalence of OPLL is of 1.9-4.3% in Japanese, 0.8-3.0% in other southeast Asian and 0.1-1.7% in North American and Europeans, suggesting a sporadic distribution [10, 11]. A recent comprehensive study from 3,161 patients in North America showed that the OPLL prevalence is various among races: 1.3% in Caucasian Americans, 4.8% in Asian Americans, 1.9% in Hispanic Americans, 2.1% in African Americans, and 3.2% in Native Americans [12]. The prevalence of OPLL in a specific location has also been reported. For example, the prevalence of thoracic OPLL (T-OPLL), mainly located at T3-T4 segments, is approximately 1.9% in Japanese, with the peak distribution of occurrence at the age of 60 years [13].

**Table 1:** Summary of OPLL susceptibility genes

References	Gene	SNPs
[75] Nishimura et al, 2012 [91] Jun and Kim, 2012	fibroblast growth factor (FGF) 2	rs1476217 rs3747676
[75] Nishimura et al, 2012 [91] Jun and Kim, 2012	fibroblast growth factor receptor (FGFR) 1	rs13317
[55] Yan et al, 2013 [54] Wang et al, 2008a [53] Wang et al, 2008b [52] Li et al, 2014 [35] Kim et al, 2014b	bone morphogenetic protein 2 (BMP2)	109T > G 570A > T 109T > G exon 3 (-726) T/C Ser37Ala (T/G) Ser87Ser (A/G)
[92] Meng et al, 2010 [56] Ren et al, 2012a [93] Furushima et al, 2002	bone morphogenetic protein 4 (BMP4)	SNP8 (C>T) rs17563 rs76335800 rs17563
[57] Ren et al, 2012b	bone morphogenetic protein 9 (BMP9)	rs7923671 rs75024165 rs34379100
[94] Chin et al, 2013a	vitamin K epoxide reductase complex subunit 1 (VKORC1)	-1639G>A
[45] Han et al, 2013 [42] Kamiya et al, 2001	transforming growth factor-beta1 (TGF-beta1)	promoter region (-509C>T) exon 1 (869T>C)
[30] He et al, 2013 [95] Nakamura et al, 1999 [28] Koshizuka et al, 2002 [29] Tahara et al, 2005	ectonucleotide pyrophosphatase phosphodiesterase 1 gene (ENPP1)	C973T IVS15-14T IVS20 A533C
[46] Jekarl et al, 2013	transforming growth factor beta receptor type 2 (TGFBR2)	455-4T-->A 571G-->A 95-35C-->T in intron 1
[38] Wei et al, 2014	collagen 17A1 (COL17A1)	rs805698 (c. G1282A, (p.G428S) rs4918079 (c.C2595T, p. R865R)
[38] Wei et al, 2014	Protein patched homolog 1 (PTCH1)	c.C3491T (p. P1164L) c.C794G (p. T265S) c.C3695T (p. P1232L)
[96] Chon et al, 2014	BH3 interacting domain death agonist (BID)	rs8190315, Ser10Gly rs2072392, Asp60Asp
[36] Kong et al, 2007 [37] Tsukahara et al, 2005 [35] Kim et al, 2014b [20] Tanaka et al, 2003	collagen 6A1 (COL6A1)	promoter (-572T), intron 32(-29) and intron 33 (+20)
[31] Koga et al, 1998	collagen 11A2 (COL11A2)	Intron 6(-4A)
[97] Kim et al, 2011 [98] Guo et al, 2014	interleukin 15 receptor, alpha (IL15RA)	rs2296139 rs2228059
[99] Chung et al, 2011	Toll-like receptor 5 (TLR5)	rs5744168, rs5744169, rs2072493, rs5744174
[100] Yan et al, 2010	20p12	rs1116867 (A/G), rs965291 (G/A)
[78] Liu et al, 2010	runt-related transcription factor 2 (RUNX2)	rs1321075 rs12333172
[101] Kim et al, 2014a	angiotensin I converting enzyme (peptidyl-dipeptidase A) 1 (ACE)	insertion/deletion (I/D) polymorphism
[27] Horikoshi et al, 2006 [102] Ogata et al, 2002	Estrogen Receptor 1 (ESR1)	rs934079 rs222848
[103] Kim et al, 2012	Estrogen Receptor 2 (ESR2)	rs1256049
[19] Matsunaga et al, 1999	HLA haplotype	N.A.
[27] Horikoshi et al, 2006	Alpha 2-Heremans-Schmid glycoprotein (AHSG)	rs2077119
[104] Numasawa et al, 1999	retinoic X receptor beta (RXRB)	T-->A substitution at nucleotide +378 (nucleotide numbering is from the start of exon 10) 3’ end +140 (an A-->T substitution) and 3’ end +561 (a nucleotide insertion, C-->CC) (numbering is from the end of exon 10)
[102] Ogata et al, 2002	interleukin 1 beta (IL-1β)	N.A.
[105] Kobashi et al, 2008	vitamin D receptor (VDR)	N.A.
[27] Horikoshi et al, 2006	transforming growth factor-beta3 (TGF-beta3)	rs226862 rs22847

OPLL can be a secondary complication in patients with several other diseases, such as hypohosphatemic rickets/osteomalacia, hypoparathyroidism and acromegaly/gigantism. Early-onset and severe OPLL usually occurs in these diseases [14, 15]. Most OPLL cases are primary (idiopathic), which are discussed in this review below. In general, OPLL is considered a multifactorial disease with both environmental and genetic factors contributing to the development. The non-genetic factors for OPLL include age, diabetes mellitus, obesity, diet, exercise and mechanical stimulation [14, 16, 17]. Plasma pentosidine levels, femoral neck bone mineral density (BMD) and diffuse idiopathic skeletal hyperostosis (DISH) are also associated with OPLL [11]. In this review, we focus on the genetic factors and signaling pathways contributing to the pathogenesis of OPLL.

## OPLL susceptibility loci

OPLL has a strong genetic predisposition. Familial OPLL cases have been reported in both Asian and Caucasian. The associated genetic loci linked to OPLL susceptibility have been identified. For example Terayama et al have reported that the prevalence of OPLL is 26% in the parents and 29% in the siblings of probands from 347 OPLL families, which is significantly higher than that in the general population [18]. Matsunaga et al found that the prevalence of OPLL is higher in the sibs sharing identical human leukocyte antigen (*HLA*) haplotypes from families of 24 OPLL patients. Later on, a non-parametric linkage analysis focusing on the HLA region revealed a significant linkage on D6S276 with OPLL [19]. Some candidate genes were identified around the marker, including collagen 11A2 (*COL11A2*) and retinoic X receptor beta (*RXRB*). Another significant linkage is D21S1903 on 21q showing collagen 6A1 (*COL6A1*) as a potential genetic factor to OPLL [20].

A genome-wide linkage study using 214 affected Japanese sib-pairs suggested OPLL-associated loci with potential linkages at 1p21, 2p22-2p24, 7q22, 16q24 and 20p12 [21]. Furthermore, a genome-wide association study (GWAS) was performed in a total of 15,000 individuals, and six susceptibility loci for OPLL were identified: 20p12.3, 8q23.1, 12p11.22, 12p12.2, 8q23.3 and 6p21.1 [22]. The genes in or near associated regions include hydroxyacid oxidase 1 (*HAO1*), r-spondin 2(*RSPO2*), eukaryotic translation initiation factor 3E (*EIF3E*), ER membrane protein complex subunit 2 (*EMC2*), coiled-coil domain containing 91 (*CCDC91*), radial spoke head protein 9 homolog (*RSPH9*) and serine/threonine kinase 38 like (*STK38L*) [22]. Further gene expression analysis showed HAO1, RSPO2 and CCDC91 had lower expression levels during early stages of chondrogenesis, while RSPH9 and STK38L showed increases in expression in osteoblasts. The authors suggested that RSPH9 and STK38L may participate in the membranous ossification process and HAO1, RSPO2 and CCDC91 play roles in the endochondral ossification process [22]. OPLL GWAS has provided essential information on chromosomal positions significantly associated with OPLL, which is far narrower than what have been defined by previous linkage studies. However, a gap between functional genomic positions and the causal genes of OPLL still needs to be bridged through more efficient target gene association studies.

## OPLL susceptibility genes and signaling

Besides linkage studies and GWAS, many target gene association studies of OPLL have been carried out over the past several decades. Numerous genes, including cytokines and growth factors, have been revealed as potential factors that contribute to the pathophysiology of OPLL, which are listed in table 1. Many candidates reside in the functional positions that have been defined by linage studies or GWAS above. In the meantime, *in vitro* and *in vivo* expression profile analysis has suggested multiple signaling pathways involved in the development and progression of OPLL, including transforming growth factor-beta (TGF-β), bone morphogenetic protein (BMP) and mechanical stress signaling. Here we focus on several major OPLL-associated candidate genes and their signaling pathways.

**Figure 1.**
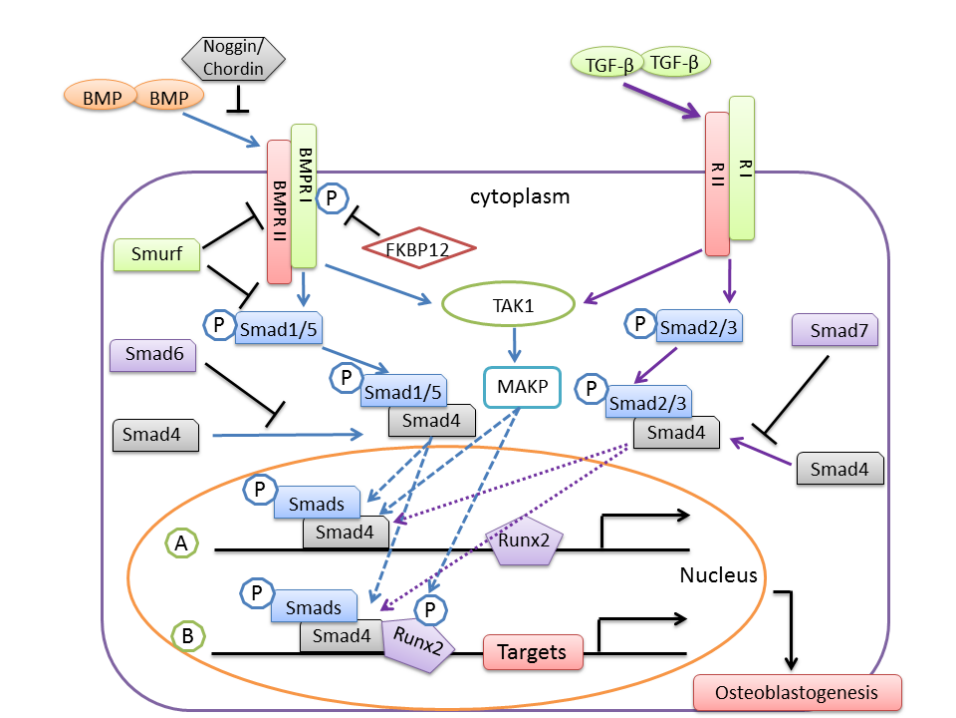
BMP/TGF-β signaling and negative regulation in osteoblastogenesis BMP or TGF-β binds to and activates their receptor type II (RII) and receptor type I (RI) and leads to subsequent phosphorylation of Smads. Activated Smads form a complex with Smad4, translocate into the nucleus and trigger transcription of Runx2. Subsequently, Smads/Smad4 associate with Runx2 to regulate target genes necessary for osteoblastogenesis. BMP/TGF-β signaling can also activate transforming growth factor beta-activated kinase 1 (TAK1) and result in activation of MAPK, leading to enhancement of Smads/Smad4 induced transcription. The negative regulations include prevention of activation of Smads by Smad6/7, inhibition of receptors activation by Smurf and FK506 binding protein 1A (FKBP12), and inhibition of BMPs binding to their receptors by Noggin/Chordin. P, phosphorylation.

### Ectonucleotide Pyrophosphatase/Phospho-diesterase 1 (*ENPP1*)

The tiptoe walking (*twy*) mice has a natural occurrence of pathological ossification of spinal ligaments, such as ossification of the posterior longitudinal ligament, enlargement of the nucleus pulposus, regenerative proliferation of annulus fibrosus cartilaginous tissues, and neovascularization and metaplasia of primitive mesenchymal cells to osteoblasts in the spinal ligaments [23, 24]. *ENPP1* was identified subsequently as the gene responsible for *twy* phenotypes, which was mutated and resulted in a stop codon in *twy* [25]. EPP1 is a type II transmembrane metalloenzyme and functions to regulate soft-tissue calcification and bone mineralization. It acts as a calcification inhibitor *via* the production of PPi [26]. Many studies have attempted to screen OPLL-associated single-nucleotide polymer-phisms (SNPs) of *ENPP1*. So far four SNPs in *ENPP1* have been found to be associated with either the development or the severity of OPLL in human. One is IVS15-14T, a T→C transition in intron 15 [27, 28], and the second is IVS20-dell11T, a T deletion at 11 nucleotides upstream of the splice acceptor site in intron 20 [29]. The remaining two involve in the coding region: A533C is an A to C change in exon 4 that alters the protein sequence from K to Q and C973T is a C to T substitution in exon 9. Patients with the IVS20-dell11T or A533C have approximately 3 times greater chance of not having disease progression after surgery, a possible predictor for post-surgery outcome [30].

### Collagen genes

Collagen participates in bone and cartilage formation and mediates the interaction between extracellular matrix components and cell surface proteins. Mutations and/or aberrant expressions of collagen genes may induce various pathological phenotypes in connective tissues. Many collagen genes are shown to be associated with OPLL, including *COL11A2*,[31-34] *COL6A1* [20, 35-37] and collagen 17A1 (*COL17A1*) [38]. COL11A2 is the type XI collagen that associates with type II collagen, the main collagenous component of cartilage. COL11A2 has been found to be significantly linked to a predisposition to OPLL [31, 34]. The retaining exon 7 together with removed exon 6 in intron 6(-4A) in *COL11A2* is speculated to play a protective role in the ectopic ossification process [32, 33]. Three different SNPs of *COL6A1* have been found to be associated with OPLL: promoter (-572T), intron 32(-29) and intron 33 (+20) [20, 35-37]. Wei et al have found two SNPs of *COL17A1*, rs805698 (c.G1282A, p.G428S) and rs4918079 (c.C2595T, p.R865R), are significantly associated with OPLL *via* a whole exome sequencing [38]. The specific roles of these SNPs of collagen genes in the pathogenesis of OPLL remain unclear, though their contributions to the formation of extracellular matrix scaffolds may facilitate endochondral ossification.

### TGF-β signaling and SNPs

The TGF-β superfamily contains over forty members, including TGF-βs, Nodal, Activin and BMPs [39]. TGF-β/BMPs signaling and their cross-talk with signaling pathways of MAPK, Wnt, Hedgehog, Notch and FGF play very crucial roles in bone formation during mammalian development [39] (Fig. 1). TGF-β is enriched in bone and cartilage that also contain a large number of target cells for TGF-β. TGF-β is critical in maintaining and expanding mesenchymal stem cells (MCS) / progenitor cells and progenitors of osteoblasts *via* autocrine and paracrine stimulations [39, 40]. Thus, the *TGF-β* genes, specifically *TGF-β1*, due to its importance in regulation of bone metabolism, are considered leading candidates in increasing individual susceptibility to OPLL [41-43]. TGF-β is present in the ossified matrix and chondrocytes of adjacent cartilaginous areas of OPLL, but not in MSCs and non-ossified ligament, suggesting it may stimulate bone formation at a later stage of ectopic ossification [44].

The 869T>C polymorphism in exon 1 of the *TGF-β1* gene has been studied extensively regarding OPLL pathogenesis, but results are contradictory. Kamiya et al. have initially reported that SNP 869T>C is a genetic determinant of predisposition for cervical OPLL, with the C allele of 869T>C representing a risk factor for genetic susceptibility to OPLL [42]. However, other three studies found no significant association between SNP 869T>C and the prevalence of OPLL in larger-scale studies [27, 43, 45]. A further stratified analysis has shown patients with the C allele of 869T>C display OPLL more frequently in the cervical, thoracic, or lumbar spine [43]. In addition, the promoter region -509C>T polymorphism of *TGF-β1* doesn’t show significant association with OPLL [45].

A large scale case-control study has shown *TGF-β3* polymorphisms of rs226862 and rs22847 are significantly associated with OPLL, among which the rs226862 polymorphism, residing deep within an intron without altering any known conserved transcription factor-binding motifs, shows the most significant association [27]. Three polymorphisms of the TGF-β receptor 2 (*TGFBR2*) gene have been found associated with OPLL: 455-4T-->A, 571G-->A and 95-35C-->T [46]. The function of these SNPs remains unclear. Though the physiological function of TGF-β indicates its potential role in the pathophysiology of OPLL, additional genetic and functional studies are necessary to reveal their roles.

### BMP signaling and SNPs

As part of TGF-β family, BMPs are the most critical growth factors that stimulate osteoblast and chondrocyte differentiation, resulting in bone and cartilage formation (Fig. 1). BMP-mediated Smad signaling plays a central role in regulating many transcriptional factors, such as Runx2, Osterix, Msx2, Dlx5/6 and Sox9 [47]. Genetic and biochemical studies indicate these transcriptional factors are essential for osteoblastogenesis and chondrogenesis [48]. The details of BMP-Smad signaling have been described in many excellent reviews [39, 49, 50]. Here we focus on the current understanding of the role of BMP signaling on OPLL pathogenesis.

Both BMP receptors and BMPs have aberrant expression patterns in OPLL tissues. BMP receptors have significant higher expression levels in the ossified ligament of OPLL than those in the non-OPLL tissue. In addition, the non-ossified segment of the PLL in OPLL specimens also expresses BMP receptors to a greater extent than that in the non-OPLL tissue [51]. BMP-2 is present in the ossified matrix and chondrocytes of adjacent cartilaginous areas of OPLL and in MSCs in the immediate vicinity of the cartilaginous areas [44]. These studies show that molecular alterations of BMP precede the development of OPLL, suggesting BMP may stimulate differentiation of mesenchymal progenitor cells and act as an initiating factor in the development of OPLL.

Many investigations have revealed the associations between *BMP* SNPs and OPLL. Several reports have showed that frequencies for the rs2273073 (T/G) and rs235768 (A/T) polymorphisms of *BMP2* are significant higher in the OPLL than those in control groups. Both SNPs are associated with the occurrence of OPLL in Chinese Han population [52-55]. The functional analysis of rs2273073 (T/G) SNP has showed this mutant aberrantly activates BMP-Smad signaling. For example, transfection of this *BMP2* mutant promotes the activation of p-Smad1/5/8, expression of Smad4 and activity of alkaline phosphatase (ALP) [55]. Uniaxial cyclic stretch promotes osteogenic differentiation and synthesis of BMP2 in the C3H10T1/2 Cells with *BMP2* gene variant of rs2273073 (T/G) [52]. However, Kim et al reported that both BMP2 SNPs showed no significant difference between OPLL and non-OPLL groups in Korean populations [35]. The diversities of these data may be resulted from variations of genetic background between these two populations. In addition, Lin’s group has characterized a total of 18 *BMP4* SNPs from 450 OPLL patients and 550 matched controls, and found that rs17563(C/T), rs76335800(A/T) and SNP8 (C>T) are the SNPs associated with increased genetic susceptibility to OPLL [56]. They also have showed that rs7923671 (T/C), rs75024165(C/T) and rs34379100 (A/C) of *BMP9* are associated with OPLL [57].

### Mechanical stress signaling

Mechanical stretch has been considered as a factor contributing to the development and progression of OPLL [58]. Numerous genes have their expression levels upregulated in response to mechanical stress, such as ALP, BMP-2, BMP-4, BPM receptors, osteopontin, Cbfa1, Type I collagen, osteocalcin, integrin β1, endothelin-1, etc [16, 59]. Mechanical stress elevates prostacyclin synthesis in ligament cells derived from OPLL patients and induces osteogenic differentiation [60]. It also regulates P2Y1 purinoceptor subtypes expression in OPLL cells and promote OPLL progression [61].

In responding to environmental mechanical stress, multiple signaling pathways are involved, including MAPK. Activation of ERK, resulting from abnormal mechanical stress, can facilitate the development of OPLL. Mechanical stress up-regulates connexin 43 (Cx43) expression in ligament fibroblasts, leading to osteoblastic differentiation [62, 63]. In a recent study, Chen et al have found Cx43, p-ERK, p-p38 MAPK and p-JNK are upregulated in OPLL as compared to non-OPLL both *in vivo* and *in vitro* [64]. The activation of these signals by mechanical stress is dependent upon Cx43. Knock-down of Cx43 blocks the ERK1/2 and p38 MAPK pathways and reverses the osteogenic effect of mechanical stress on ligament fibroblasts. Therefore, Cx43 functions to promote the osteoblastic differentiation of ligament fibroblasts partly *via* the activation of ERK1/2 and p38 MAPK signals [64]. Another group has found that the cultured OPLL fibroblasts exhibit osteogenic characteristics and show increases in expressions of p-EKR, osteocalcin (OCN), ALP and collagen 1 (COL1) as compared to non-OPLL fibroblasts. Knockdown of EKR in OPLL fibroblasts inhibits expression of OCN, ALP and COL1, indicating that p-ERK-mediated ER stress might be involved in the development of OPLL [65].

Vimentin, a type III intermediate filament protein, is also involved in responding to mechanical stretch in OPLL cells. Overexpression of vimentin in osteoblasts results in decrease in ALP activity and expression of osteoblast markers, and delay of mineralization, leading to inhibition of osteoblast differentiation.[66] Expression of vimentin can be downregulated by mechanical stress, which leads to increase in expressions of OCN, ALP and COL 1 in OPLL cells. Thus vimentin may play an important role in the progression of OPLL through the induction of osteogenic differentiation in OPLL by its down-regulation [67].

### Other potential pathways

A microarray study of 1536 genes in OPLL as compared to control showed wound healing is the most activated signaling occurred in OPLLs with the upregulations of platelet-derived growth factor-B (PDGFB) and peroxiredoxin 2 (PRDX2). The Toll-like receptor signaling pathway had the most significant changes in OPLL, with upregulations of some key members in Toll-like pathway such as Toll-like receptor 1 (TLR 1), TLR5, TLR7, and mitogen-activated protein kinases 10 (MAPK10) and phosphoinositide-3-kinase (PI3K), regulatory subunit 1 (PIK3R1), suggesting the crucial roles of PI3K in the development of OPLL [68].

## MSCs and OPLL

MSCs are multipotent progenitor cells that can differentiate into a variety of cell types, including osteoblasts and chondrocytes. MSCs have been reported to play important roles in pathogenic development of several ossification process, such as fibrodysplasia ossificans progressiva (FOP) [69], ectopic ossification following burn injury[70] and aortic valve calcification [71]. The initial attempt to isolate MSCs from human spinal ligaments was made by Asari et al. They found these MSCs have potentials to differentiate into osteogenic, adipogenic or chondrogenic cells with expressions of surface markers of CD34, 73, 90 and 105 but not CD45. They located in the collagenous matrix of the ligament and perivascular areas [72]. Further study showed that MSCs with perivascular residing expressed pericytes marker α-smooth muscle actin (α-SMA) but not endothelial marker CD31, thus was a subpopulation of pericytes. This is coincident with the theory that a subset of pericytes was MSCs. The ossified ligamentum flavum (OLF) showed a significant higher quantity of MSCs around blood vessels and within collagenous matrix as compared to non-OLF samples [73]. OPLL-derived MSCs showed significantly higher osteogenic differentiation potential and *in vitro* increases in activity of ALP and expressions of BMP2, runt-related transcription factor 2 (Runx2) and ALP than those from non-OPLL patients. The author suggested that an upregulation of osteogenic differentiation potential of OPLL-derived MSCs could be a causal factor to the ossification in spinal ligaments [74].

Many transcriptional factors regulate osteogenesis and chondrogenesis from MSCs, among which Msx2, Sox9, Runx2 and Osterix are the central components for bone and cartilage development [75]. As a key player in osteoblast differentiation, Runx2 regulates expressions of all major osteoblasts-specific genes, including ALP, type I collagen, osteopontin and OCN, *via* binding to the osteoblast-specific *cis*-acting element 2 in their promoters. Runx2 have been shown to be linked to OPLL by many lines of evidence. Runx2 expression in OPLL cells is significantly higher than that in non-OPLL [16]. Inhibition of both Runx2 and angiopoietin-1, a downstream of Runx2 in OPLL cells and osteoblasts, results in a complete blockage of aggrecan-1, suggesting that Runx2/angiopoitin-1 plays an important role in ectopic calcification [76]. As a upstream regulator of Runx2, promyelotic leukemia zinc finger (PLZF) is upregulated in OPLL cells and facilitates expression of osteoblasts-specific genes in MSCs, thus promoting the ossification of spinal ligament cells in OPLL patients [77]. In addition, *Runx2* SNPs, rs1321075 and rs12333172, have been linked to increases in incidence of OPLL and OLF [78]. Runx2 has been shown to induce hypertrophy of chondrocytes through induction of indian hedgehog (Ihh) [79]. Sugita et al have shown that the MSCs in OPLL tissues have expressions of Ihh, Sox9 and parathyroid-related peptide hormone (PTHrP). Ihh and Sox9 are highly expressed in proliferating chondrocytes and PTHrP is strongly expressed in hypertrophic chondrocytes. Their expressions are significantly higher in OPLL-derived cells as compared to non-OPLL in culture. These lines of evidence suggest Ihh together with Runx2 and Sox9 regulates chondrocytes differentiation in enchondral ossification process in OPLL [80].

## OPLL Biomarkers

Sclerostin and dickkopf-1(DKK1) are Wnt/β-catenin signal antagonists that play an important role in bone formation. Sclerostin level reflects age-related changes in bone mass and turnover rate [81]. The secretion of sclerostin increases from individual osteocytes with aging [82]. Deletion of a single allele of the Dkk1 gene leads to an increase in bone formation and bone mass [83]. Serum sclerostin levels in the male OPLL subjects group are significantly higher than those in the control group, which is positively correlated with age and bone mineral density of total hip (TH-BMD). Serum sclerostin and DKK1 levels are negatively correlated in male OPLL subjects. Systemic secretion of sclerostin also increases with advancing age and with higher bone mass in male OPLL subjects [84].

Serum carboxyterminal propeptide of human type 1 procollagen (PICP) and intact osteocalcin are significantly increased in OPLL patients, with a significant correlation to BMD [85]. Serum concentrations of bone formation markers including OCN and PICP show a positive correlation to the OPLL, reflecting the activity of general ectopic bone formation in patients [86]. Serum insulin levels are significantly associated with the extent of OPLL [87]. Serum leptin concentrations corrected for body mass index correlate positively with the number of vertebrae with OPLL involvement in female OPLL subjects, suggesting hyperleptinemia in combination with hyperinsulinemia contributes to the development of heterotopic ossification of the spinal ligament [88].

A proteomic analysis showed carbonic anhydrase I, NAD(P) dependent steroid dehydrogenase-like, billiverdin reductase B and alpha-1 collagen VI were down-regulated in OPLL ligaments, while osteoglycin (OGN) and nebulin-related anchoring protein are up-regulated in ligaments, as compared to those in non-OPLL ligaments [89]. OGN is known to regulate type I collagen fibrillogenesis *via* BMP-1 signaling [90]. The detailed functions of these proteins in pathogenesis of OPLL are unknown.

## Summary and Future Perspective

Many attempts have been made to identify both genetic and environmental factors that cause OPLL. The recent GWAS analysis based on large-scale samples have significantly narrowed the functional positions on the chromosomes that may harbor the causal genes of OPLL. However, more efficient methods and statistical analysis are required to identify the target genes from these regions. Although a number of OPLL susceptibility genes have been revealed with appearance of specific SNPs in OPLL specimens for over several decades, most studies are based on small sample sizes and small number of examined sequence variants. Thus, a systematic SNP characterization in OPLL tissues based on large scale samples using whole genome or exome next generation sequencing method will be helpful to close this gap. In addition, lack of further functional characterizations of these SNPs makes the genetic association studies insufficient. Whether the SNPs identified to be associated with OPLL are non-functional consequences from the development and progression of OPLL or the causes to the disease remains to be determined.

**Figure 2.**
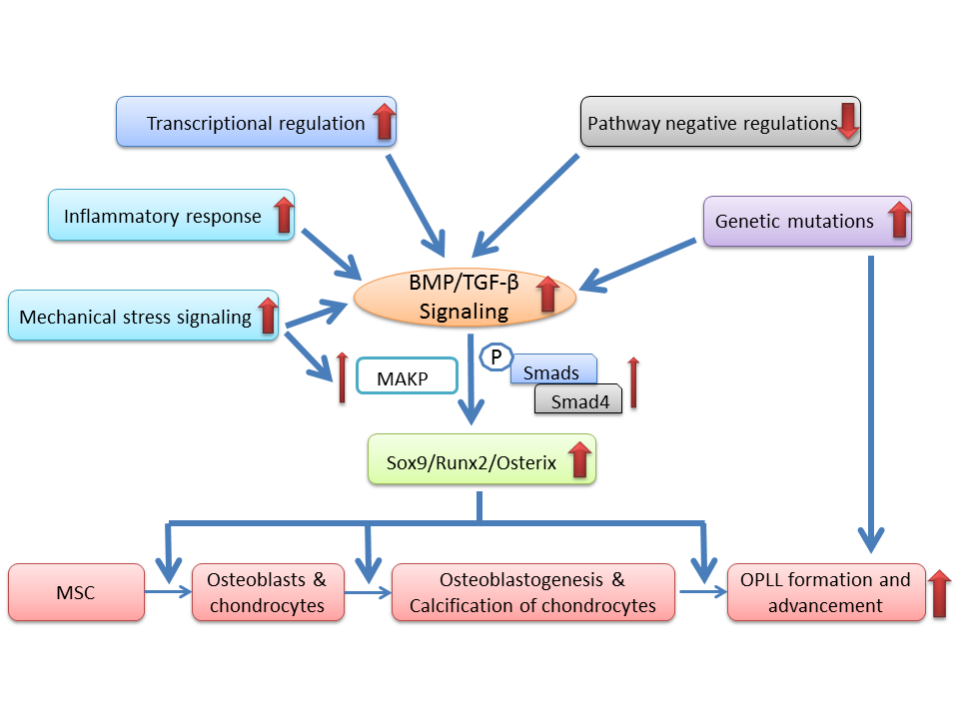
Hypothetic pathogenesis of OPLL. Aberrant activation of BMP and TGF-β signaling plays a central role in development of OPLL Many pathological alterations, including mechanical stress, inflammatory response, transcriptional and pathway negative regulations, and genetic mutations, can cause activation of BMP and TGF-β signaling. As a result, Smads/Smad4 and MAPK are upregulated and transcriptions of Sox9, Runx2 and Osterix are increased, which modulates the differentiation and proliferation of MSCs, osteoblasts and chondrocytes and ultimately causes OPLL formation and advancement.

The biochemical and cell biological data on characterizing OPLL-derived MSCs and aberrant signaling pathways such as BMP, TGF-β and MAPK have begun to provide new revenues to tackle the question of OPLL pathogenesis. The prominent perivascular locations of MSCs in OPLL tissues suggest MSCs are blood vessel origin which may determine the fate of heterotopic ossification during their differentiation. This line of new evidence has offered fresh insights on the molecular mechanisms underlying OPLL. However, how the aberrant signaling recruits MSCs from pericytes and transits them into heterotopic ossification are the most critical questions yet to be answered, which should be one of the future directions and foci. In summary, we propose that the aberrant activation of BMP and TGF-β signaling play a central role in the pathogenesis of OPLL, which can be caused by mechanical stress, radicular pain-induced inflammatory response, transcriptional and pathway negative regulations, and genetic alterations (Fig. 2). Subsequently, the activation of Smads/Smad4 and MAPK upregulates many crucial osteoblastogenic and chondrogenic transcriptional factors, such as Sox9, Runx2 and Osterix, which results in modulation of differentiation and proliferation of MSCs, osteoblasts and chondrocytes and ultimately causes development and progression of OPLL. Genetic mutations in many genes also directly participate in the pathogenic process of progressive ectopic ossification *via* BMP/TGF-β independent pathways (Fig. 2).
